# Effects of single and repeated bouts of flywheel exercise on jump performance and muscle damage in athletes and non athletes

**DOI:** 10.1186/s13102-023-00785-2

**Published:** 2024-01-02

**Authors:** Takashi Shimizu, Yosuke Tsuchiya, Hisashi Ueda, Shigeki Izumi, Eisuke Ochi

**Affiliations:** 1 Faculty of Health Care, Teikyo Heisei University, 2-51-4, Higashiikebukuro, Toshima, Tokyo 170-8445 Japan; 2 Graduate School of Sports and Health Studies, Hosei University, 4342, Aihara-Cho, Machida, Tokyo 194-0298 Japan; 3 Center for Liberal Arts, Laboratory of Health and Sports Sciences, Meiji Gakuin University, 1518, Kamikurata-Cho, Totsuka, Yokohama, Kanagawa 244-8539 Japan; 4 Faculty of Health and Medical Science, Teikyo Heisei University, 4-1, Uruidominami, Ichihara, Chiba 290-0193 Japan; 5 Faculty of Bioscience and Applied Chemistry, Graduate School of Sports and Health Studies, Hosei University, 3-7-2 Kajino, Koganei, Tokyo 184-8584 Japan

**Keywords:** Muscle damage, Acute response, Jump performance, Basketball player, Flywheel exercise, Repeated-bout effect

## Abstract

**Background:**

Although recent studies have investigated the effects of flywheel (FW) training on muscle function, the effects of transient FW exercise on jump performance in athletes are unknown. This study examined the effects of single and repeated bouts of FW squat exercises on jump performance and muscle damage in male collegiate basketball players.

**Methods:**

The participants were 10 healthy college-age men (nonathletes) and 11 male basketball players (athletes). The intervention involved 100 squat exercises (10 repetitions × 10 sets) using an FW device. To examine the repeated-bout effects, the protocol was conducted again after a 2-week interval. Squat jumps, countermovement jumps, drop jumps, and rebound jumps were evaluated as jump performance, while isometric maximal voluntary contraction (MVC) torque in knee extension, muscle soreness, range of motion, thigh circumference, muscle thickness, and echo intensity were evaluated as markers of muscle damage. Measurements were taken at baseline, immediately after exercise, 24 h later, and 72 h later.

**Results:**

The jump performance of nonathletes decreased after exercise (*p* < 0.05), while that of the athletes did not. The results were similar for muscle soreness. MVC torque decreased significantly after the first exercise in both groups (*p* < 0.05) and was significantly lower in the nonathletes versus athletes. Significant repeated-bout effects were found for muscle soreness in nonathletes but not athletes.

**Conclusions:**

These results suggest that a single bout of FW exercise reduces jump performance in male nonathletes but not basketball players.

## Background

Eccentric contractions (ECCs) are known to cause higher torque output than concentric contractions (CONs) and isometric contractions. ECCs are also widely known to induce delayed-onset muscle soreness and musculoskeletal damage such as muscle swelling and reduce muscle function and flexibility and neurological function [1–3]. Therefore, it is important to elucidate the characteristics of muscle damage induced by ECC exercise.

ECC exercise, bearing a large load during the eccentric phase, reportedly induces muscular hypertrophy [4], increases muscular strength, and improves jump and sprint performance [1, 5]. Several recent reviews have clearly shown the effectiveness of training using a flywheel (FW) [6–8]. Such FW training makes use of the load due to inertia when a disk-shaped weight, an FW, is rotated with acceleration and deceleration, which can repeatedly manifest greater force during the eccentric versus concentric phase [9]. Of particular interest are the numerous reports of the efficacy of FW training for increasing muscular power and jump performance in athletes [10–15].

There has been little research on muscle damage induced by acute FW exercise, and much remains uncertain in this area. Carmona et al. (2015) [16] showed that, in healthy and recreationally active young men, FW exercise involving acute squats resulted in delayed-onset muscle soreness and increased serum creatine kinase levels. Similarly, Coratella et al. (2016) [17] reported that, in untrained males, FW exercise involving squats resulted in delayed-onset muscle soreness, increased serum CK, and decreased isometric maximal voluntary contraction (MVC). The repeated-bout effect was also investigated in this study [17]. A second FW exercise after a 4-week interval significantly suppressed the MVC decrease, delayed-onset muscle soreness, and serum CK increase compared with the first bout [17]. However, no reports have discussed the effects of acute FW exercise on muscle damage in athletes.

Jump performance is a crucial factor in sports such as basketball and volleyball because they require frequent jumping [18, 19]. Indeed, there is an association between countermovement jump (CMJ) performance and competition level among basketball players [20]. Therefore, we hypothesized that elucidating the effects of FW exercise on jump performance and muscle damage in basketball players would provide important information to improve sports performance and training programs. Therefore, the present study aimed to examine (i) the acute effect of squat exercises using an FW device on jump performance and muscle damage in basketball players and nonathletes; and (ii) the repeated-bout effect of jump performance and muscle damage caused by FW exercise. Since basketball players routinely perform jump movements [20, 21], we hypothesized that the jump performance decrease, muscle damage severity, and repeated-bout effect degree would be smaller in basketball players than nonathletes.

## Methods

### Participants

The participants included 10 healthy untrained men (nonathletes; age, mean ± standard deviation [SD], 19.5 ± 1.1 years; height, 171.0 ± 6.6 cm; body mass, 67.9 ± 9.7 kg) and 11 basketball players (athletes; age, 20.3 ± 0.9 years; height, 183.1 ± 6.9 cm; body mass, 76.7 ± 8.5 kg). The nonathletes had not performed any regular resistance training for at least 1 year prior to participating in the study. The athletes were collegiate basketball players competing in Japan’s Kanto College Basketball Federation; all had at least 6 years of playing experience. They completed five 2-h practices and one game each week during the season. All of the players were familiar with resistance training, which they performed once a week during the season, but none had experience using FW devices. In addition, none of the players had sustained a severe injury in the 2 years before the study, and none reported having any diseases or taking any medication during the intervention. All participants were asked to avoid any interventions, such as massage and medication intake, during the experimental period. Each was given a detailed explanation of the study protocol before participating and provided written informed consent.

The study was approved by the Teikyo Heisei University Ethical Committee Involving Human Subjects (ID: 2022–009-2). The sample size was determined by a power analysis (G*power version 3.1.9.4, Heinrich-Heine University, Dusseldorf, Germany) by setting the effect size as 0.25, an α level of 0.05, and a power (1-β) of 0.80 for the intergroup comparison, which showed that at least 20 participants were necessary.

### Protocol

The study involved two bouts of FW exercise spaced 2 weeks apart (FW1 and FW2). One week before the start of the experiment, both groups performed FW exercises to familiarize themselves with the FW device (kBOX4 Active Advanced System; Exxentric AB, Stockholm, Sweden) [17, 22]. To investigate muscle damage, the participants were tested at baseline (Pre) and immediately after (Post), 24 h after (Day 1), and 72 h after (Day 3) the exercise bout for a total of four measurements. Then, to examine the magnitude of the repeated-bout effect, the same protocol was repeated 2 weeks later. To investigate the effects on jump performance and muscle damage caused by enhanced eccentric squats with the FW device, squat jumps (SJ), countermovement jumps (CMJ), drop jumps (DJ), rebound jumps (RJ), MVC torque, muscle soreness, range of motion (ROM), circumference, muscle thickness, and echo intensity were measured.

### Eccentric exercise

The intervention consisted of 10 sets × 10 repetitions of squats using the FW device with an inertia load of 0.05 kg ⋅ m^2^. The exercise protocol was determined based on previous research by Coratella et al. [17]. The participants were instructed to perform the concentric phase as quickly as possible and the eccentric phase until the knee angle was approximately 90°. Each was requested to complete the concentric (1 s) and eccentric (2 s) cycle in a total of 3 s. Peak and mean power for each repetition (eccentric and concentric) were recorded using the software (kMeter App) preinstalled on the FW device. In addition, all participants were strongly and regularly encouraged to maximally perform each repetition. A 60-s rest period was provided between sets.

### Rate of perceived exertion

The rate of perceived exertion (RPE) was measured using a psychophysical category scale, with the participant rating the strength of his perception from 6 (“no exertion at all”) to 20 (“extremely strong exertion”). RPE measurements were recorded immediately upon the completion of each set of FW exercises.

### Jump performance

Jump performance was assessed for the SJ, CMJ, DJ, and RJ to evaluate the power output and stretch–shortening cycle of the lower limb muscles. The participant performed each jump on a jump mat (Multi Jump Tester II; DKH Inc., Tokyo, Japan) connected to a computer. The participants placed their hands on their hips to prevent arm swinging and were instructed to jump as high as possible. Their flight and contact time were recorded during the jump, while the jump height was calculated from the flight time using the following formula: jump height (cm) = 1/8 (flight time) × gravitational acceleration (= 9.81 m/s^2^). The participants performed the DJ by jumping off of a box (20- and 40-cm heights) and landing on the jump mat and were instructed to perform a maximal vertical jump with minimal contact time. The RJ was performed five times, with the participants instructed to perform a maximal vertical jump with minimal contact time. The DJ and RJ indexes were calculated as jump height divided by contact time. The SJ, CMJ, and DJ tests were performed twice each with a 2-min rest between them, while the RJ was performed only once. The highest jump height and the RJ and DJ indexes for each participant were used in the analysis.

### Isometric MVC torque

For the measurement of MVC torque of knee extension, the participants performed the 3-s maneuvers twice with a 60-s rest between them. The participants performed knee extension in the dominant leg, and the MVC torque was measured using a peak dynamometer (Primus RS; BTE Technologies, Hanover, MD, USA) [23]. Device calibration and gravity correction were performed according to the manufacturer’s protocols. The participants were tested while sitting in a chair with a backrest. The anatomical axis of rotation of the knee joint was aligned with the dynamometer axis, and the pad of the tool was positioned centrally at the lower part of the shin (i.e., the tibia). The knee was kept at 90° of flexion, the hip in neutral rotation and abduction, and the foot in plantar flexion. The hands were placed on the abdomen, while the trunk, hips, and mid-thigh were stabilized against the chair with Velcro straps. The participants were instructed to extend their knees (exert pressure upward on the pad) and perform the MVC.

### Muscle soreness

Muscle soreness was assessed using a digital muscle stiffness instrument (NEUTONE TDM-NA1; Try-All Corp., Chiba, Japan) to apply pressure to the vastus lateralis, vastus medialis, and rectus femoris. The pressure was applied perpendicular to the halfway point between the femoral and lateral condyles of each muscle. All tests were conducted by the same investigator, who had practiced the procedure many times with different participants. Muscle soreness was assessed using a 10-cm visual analog scale in which 0 was “no pain” and 10 was “the worst pain imaginable.” The participants were instructed to indicate their pain sensation accordingly. Subsequently, the experimenter measured the distance between the left margin and the participant’s answer and used it in the data analysis.

### Range of motion

ROM was measured using a goniometer (Takase Medical, Tokyo, Japan). Flexion was measured when the participant attempted to maximally flex the knee joint of the dominant leg to touch his hip with his heel while keeping the knee joint aligned with the standing leg. Extension was measured when the participant attempted to maximally extend the knee joint of the exercised leg. ROM was calculated by subtracting the flexion from the extension of the knee joint.

### Circumference

When each participant stood with his feet approximately 10 cm apart and his body weight evenly distributed on the feet, the perimeter distance of the thigh perpendicular to the long axis of the femur at the mid-trochanteric–tibial level was measured.

### Muscle thickness and echo intensity

B-mode ultrasound images of the vastus lateralis, vastus medialis, and rectus femoris muscles were captured using an ultrasound device (SONIMAGE HS1; Konika Minolta, Tokyo, Japan), and the probe was placed at the mid-trochanter–tibial level at the same position marked for the circumference measurement. The same gain and contrast were used throughout the experimental period. The transverse images of each muscle were transferred to a computer as bitmap files (.bmp) and analyzed. The thicknesses of the vastus lateralis, vastus medialis, and rectus femoris were manually calculated by tracing using image analysis software (ImageJ; National Institutes of Health, Bethesda, MD, USA). The mean muscle echo intensity of the region of interest (20 × 20 mm) was calculated using the same software to generate a grayscale histogram (0, black; 100, white) for the region as described previously [24].

### Statistical analysis

All statistical analyses were performed using SPSS Statistics software version 22.0 (IBM Corp., Armonk, NY, USA). Values are expressed as mean ± standard deviation. All data were confirmed as normally distributed using the Shapiro–Wilk test. Changes in peak torque and RPE during the ECCs were compared using one-way repeated-measures analysis of variance (ANOVA). MVC torque, jump performance, muscle soreness, ROM, circumference, muscle thickness, and echo intensity were compared between the athlete and nonathlete groups by two-way repeated-measures ANOVA. In addition, *t*-tests were performed to compare the peak values minus the value of pre (Δ) for each measure with FW1 and FW2. The average change in muscle soreness was analyzed for the vastus lateralis, vastus medialis, and rectus femoris. A significant main effect or interaction was found; Bonferroni’s correction was performed for post hoc testing, including the difference from baseline. To demonstrate the effect sizes, Cohen’s d was calculated for the *t*-tests, and the partial eta squared (η^2^) was calculated for the ANOVA [25]. A general guideline for interpreting Cohen’s d is as follows: small (0.20), medium (0.50), and large (0.80). In addition, a general guideline for interpreting η^2^ is as follows: small (0.01), medium (0.06), and large (0.14). Values of *p* < 0.05 were considered statistically significant.

## Results

### Isometric MVC torque, jump performance, and peak power

The baseline MVC torque, jump performance, and peak power are shown in Table 1. The mean MVC torque was significantly higher for athletes (126.3 ± 16.2 Nm) than for nonathletes (81.7 ± 12.4 Nm) (*p* < 0.05, d = 3.07). In terms of jump performance, mean SJ and CMJ were significantly higher in athletes (38.4 ± 3.9 cm and 41.8 ± 3.2 cm, respectively) than in nonathletes (33.4 ± 4.8 cm vs. 37.2 ± 3.9 cm) (both *p* < 0.05; SJ, d = 1.14; CMJ, d = 1.13). Similarly, mean peak power in FW exercises in the concentric phase was higher in athletes than in nonathletes (886.0 ± 206.7 W vs. 595.7 ± 232.8 W) (*p* < 0.05, d = 1.32). However, the mean peak power in the FW exercises in the eccentric phase did not differ significantly between groups (905.6 ± 313.0 W vs. 709.1 ± 273.5 W).

**Table 1:** Physiological characteristics at baseline

	Nonathletes *n* = 10	Athletes *n* = 11	*P* value
Age, years	19.5 ± 1.1	20.3 ± 0.9	0.09
Height, cm	171.0 ± 6.6	183.1 ± 6.9	< 0.01
Weight, kg	67.9 ± 9.7	76.7 ± 8.5	0.04
MVC torque, Nm	81.7 ± 12.4	126.3 ± 16.2	< 0.01
Squat jump, cm	33.4 ± 4.8	38.4 ± 3.9	0.02
Counter movement jump, cm	37.2 ± 3.9	41.8 ± 3.2	< 0.01
Concentric peak power, W	595.7 ± 232.8	886.0 ± 206.7	< 0.01
Eccentric peak power, W	709.1 ± 273.5	905.6 ± 313.0	0.14

*MVC* maximum voluntary contraction

### Eccentric exercises

#### Power

The peak and average powers during the FW exercise (10 sets × 10 repetitions) in the concentric and eccentric phases are shown in Table 2. In both FW1 and FW2, athletes had a significantly higher peak and mean power in the concentric and eccentric phases than nonathletes.

**Table 2:** Power exerted during the flywheel exercise

	FW1			FW2		
	Nonathletes	Athletes	*P* value	Nonathletes	Athletes	*P* value
CON peak power, W	488.3 ± 151.9	823.6 ± 218.1	< 0.01	685.7 ± 193.5	1020.6 ± 205.4	< 0.01
ECC peak power, W	623.4 ± 289.4	859.6 ± 229.7	0.04	853.5 ± 277.2	1172.7 ± 289.3	0.01
CON average power, W	399.1 ± 125.6	693.4 ± 202.5	< 0.01	559.8 ± 169.4	871.5 ± 186.5	< 0.01
ECC average power, W	486.1 ± 224.9	706.4 ± 205.7	0.02	689.7 ± 225.3	974.6 ± 257.9	< 0.01

*CON* concentric contractions, *ECC* eccentric contractions, *FW1* first flywheel exercise session, *FW2* second flywheel exercise session

### Rate of perceived exertion

The RPE values for each set are shown in Table 3. In FW1, the peaks for nonathletes and athletes were 19.2 ± 1.5 and 19.6 ± 0.9, respectively. In FW2, the peaks for nonathletes and athletes were 19.5 ± 1.0 and 19.5 ± 1.3, respectively. There were no statistically significant intergroup differences.

**Table 3:** Rate of perceived exertion during flywheel exercise

		FW1		FW2	
		Nonathletes	Athletes	Nonathletes	Athletes
RPE	1 set	12.9 ± 2.7	13.2 ± 2.7	9.9 ± 2.5	12.9 ± 2.9
	2 sets	13.7 ± 2.1	14.2 ± 2.3	12.3 ± 3.4	14.2 ± 2.1
	3 sets	15.1 ± 2.2	14.6 ± 2.5	13.5 ± 3.1	15.3 ± 2.1
	4 sets	16.3 ± 2.5	15.5 ± 2.7	14.7 ± 3.5	16.1 ± 2.4
	5 sets	16.6 ± 2.1	16.4 ± 2.6	16.1 ± 3.3	17.0 ± 2.0
	6 sets	17.3 ± 1.8	17.3 ± 2.5	17.1 ± 3.2	17.5 ± 2.0
	7 sets	18.3 ± 2.5	17.8 ± 2.2	18.0 ± 2.4	18.1 ± 1.8
	8 sets	19.0 ± 1.5	18.6 ± 1.7	18.7 ± 2.3	18.6 ± 1.7
	9 sets	19.0 ± 1.6	19.1 ± 1.4	18.9 ± 2.2	19.0 ± 1.4
	10 sets	19.2 ± 1.5	19.6 ± 0.9	19.5 ± 1.0	19.5 ± 1.3

*RPE* rate of perceived exertion

### Jump performance

#### Squat jump

**Fig. 1:**
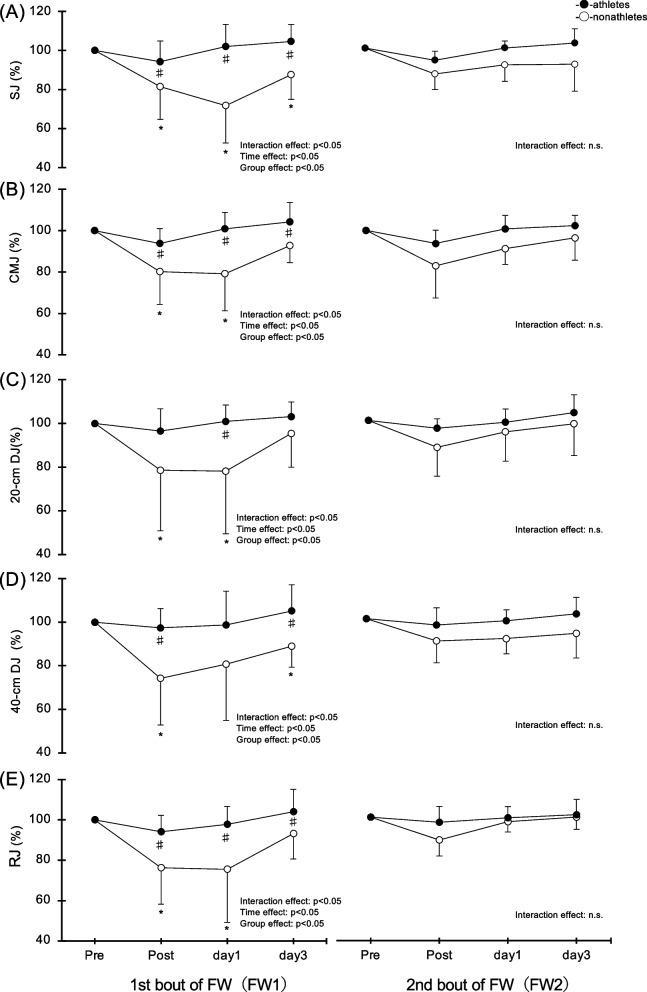
Changes (mean ± SD) in squat jump (**A**), countermovement jump (**B**), 20-cm drop jump height (**C**), 40-cm drop jump height (**D**), and rebound jump height (**E**) measured before (pre) and immediately after (post) the first (FW1) and second (FW2) flywheel exercise and 1 and 3 days after in the nonathletes versus athletes groups. *p < 0.05 for the difference from the pre-exercise value in the nonathletes group. ♯*p* < 0.05 for the difference between nonathletes and athletes groups

In FW1, there was a significant interaction for SJ (*p* < 0.05, η^2^ = 0.04; Fig. 1A). Nonathletes showed a significant decrease at Post (27.2 ± 6.6 cm), Day 1 (24.1 ± 8.1 cm), and Day 3 (29.3 ± 6.6 cm) after exercise compared with Pre (33.4 ± 4.8 cm) (all *p* < 0.05). Furthermore, SJ was significantly greater in athletes than nonathletes at Post (athletes: 36.0 ± 4.5 cm), Day 1 (athletes: 39.0 ± 5.1 cm), and Day 3 (athletes: 40.0 ± 4.5 cm) (all *p* < 0.05). No significant interactions were found in FW2. Squat jump height remained unchanged in athletes.

### Countermovement jump

In FW1, there was a significant interaction for CMJ (*p* < 0.05, η^2^ = 0.22; Fig. 1B). Nonathletes showed a significant decrease at Post (30.1 ± 7.7 cm) and Day 1 (29.5 ± 8.1 cm) after exercise compared with Pre (37.2 ± 3.9 cm) (both *p* < 0.05). Furthermore, the mean CMJ was significantly greater in athletes than in nonathletes at Post (athletes: 39.1 ± 3.7 cm), Day 1 (athletes: 42.1 ± 3.7 cm), and Day 3 (nonathletes: 34.6 ± 6.0; athletes: 43.5 ± 4.6 cm) (all *p* < 0.05). No significant interactions were found in FW2.

### Twenty-centimeter DJ

In FW1, there was a significant interaction for the 20-cm DJ (*p* < 0.05, η^2^ = 0.05; Fig. 1C). Nonathletes showed a significant decrease at Post (21.2 ± 8.0 cm) and Day 1 (20.4 ± 6.9 cm) after exercise compared with Pre (26.8 ± 4.6 cm) (both *p* < 0.05). Furthermore, 20-cm DJ was significantly greater in athletes than nonathletes on Day 1 (athletes: 34.8 ± 5.6 cm) (*p* < 0.05). No significant interactions were found in FW2.

### Forty-centimeter DJ

In FW1, there was a significant interaction for the 40-cm DJ (*p* < 0.05, η^2^ = 0.04; Fig. 1D). Nonathletes showed a significant decrease at Post (20.9 ± 7.5 cm) and Day 3 (24.5 ± 4.1 cm) after exercise compared with Pre (27.7 ± 4.7 cm) (both *p* < 0.05). Furthermore, the mean 40-cm DJ was significantly greater in athletes than nonathletes at Post (athletes: 34.4 ± 4.5 cm) and Day 3 (athletes: 37.1 ± 4.9 cm) (both *p* < 0.05). No significant interactions were found in FW2.

### Rebound jump

In FW1, there was a significant interaction for RJ (*p* < 0.05, η^2^ = 0.15; Fig. 1E). Nonathletes showed a significant decrease at Post (23.0 ± 6.5 cm) and Day 1 (22.4 ± 8.4 cm) after exercise compared with Pre (30.2 ± 5.7 cm) (both *p* < 0.05). Furthermore, the mean RJ was significantly greater in athletes than nonathletes at Post (athletes: 33.6 ± 3.5 cm), Day 1 (athletes: 34.9 ± 3.7 cm) and Day 3 (nonathletes: 27.8 ± 4.4 cm, athletes: 37.0 ± 3.3 cm) (all *p* < 0.05). No significant interactions were found in FW2.

### Isometric MVC torque of knee extension

**Fig. 2:**
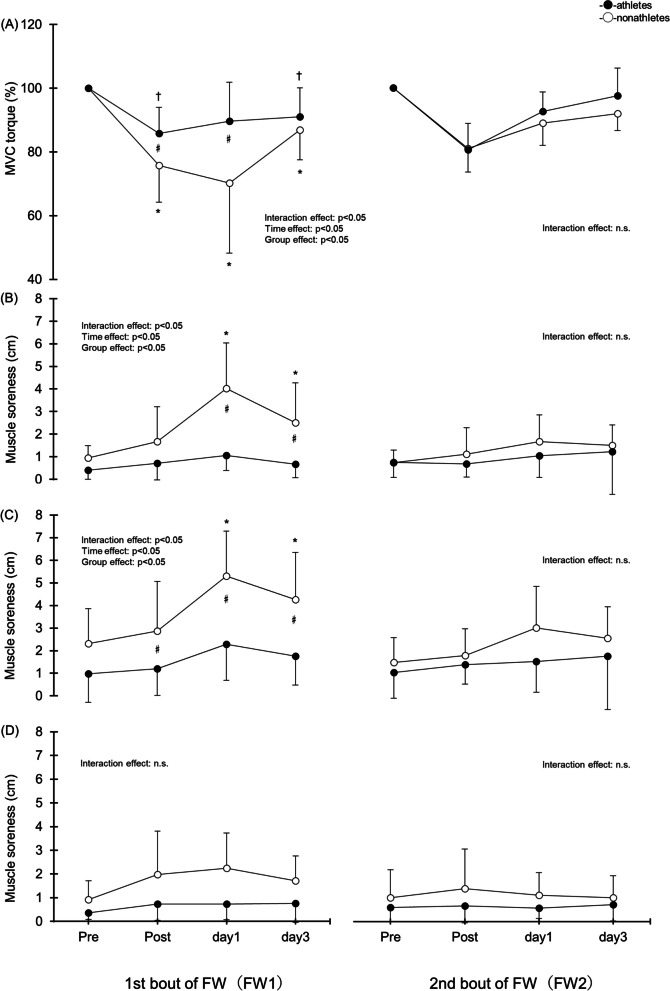
Changes (mean ± SD) in MVC torque (**A**), muscle soreness in the vastus lateralis (**B**), vastus medialis (**C**), and rectus femoris (**D**) measured before (pre) and immediately after (post) the first (FW1) and second (FW2) flywheel exercise and 1 and 3 days after in the nonathletes versus athletes groups. *p < 0.05 for the difference from the pre-exercise value in the nonathletes group. †*p* < 0.05 for the difference from the pre-exercise value in the athletes group. ♯*p* < 0.05 for the difference between nonathletes and athletes groups

In FW1, there was a significant interaction for MVC torque (*p* < 0.05, η^2^ = 0.11; Fig. 2A). Nonathletes showed a significant decrease at Post (62.2 ± 15.1 Nm), Day 1 (56.3 ± 18.3 Nm), and Day 3 (71.2 ± 14.5 Nm) after exercise compared with Pre (81.7 ± 12.4 Nm) (all *p* < 0.05). In addition, athletes showed a significant decrease at Post (108.9 ± 19.7 Nm) and Day 3 (115.5 ± 21.0 Nm) after exercise compared with Pre (126.3 ± 16.2 Nm) (both *p* < 0.05). Furthermore, there was a significant difference between the groups at Post and Day 1 (athletes: 114.2 ± 24.8 Nm) (both *p* < 0.05). No significant interactions were found in FW2.

### Muscle soreness

In FW1, there was a significant interaction for muscle soreness in the vastus lateralis (*p* < 0.05, η^2^ = 0.30, Fig. 2B) and vastus medialis (*p* < 0.05, η^2^ = 0.31, Fig. 2C). In the vastus lateralis, nonathletes showed significant increases on Day 1 (4.0 ± 2.0 cm) and Day 3 (2.5 ± 1.8 cm) compared with Pre (1.0 ± 0.5 cm) (both *p* < 0.05). Furthermore, there was a significant difference between the groups on Day 1 (athletes: 1.1 ± 0.7 cm) and Day 3 (athletes: 0.7 ± 0.6 cm) (both *p* < 0.05). In the vastus medialis, nonathletes showed a significant increase on Day 1 (5.3 ± 2.0 cm) and Day 3 (4.3 ± 2.1 cm) compared with Pre (2.3 ± 1.5 cm) (both *p* < 0.05). Furthermore, there was a significant difference between the groups at Post (nonathletes: 2.9 ± 2.2 cm, athletes: 1.2 ± 1.2 cm), Day 1 (athletes: 2.3 ± 1.6 cm), and Day 3 (athletes: 1.8 ± 1.3 cm) (all *p* < 0.05). No significant interaction was found in the rectus femoris (Fig. 2D) in FW1, and no significant interactions were found in any of the muscles in FW2.

### ROM, circumference, muscle thickness, and echo intensity

**Fig. 3:**
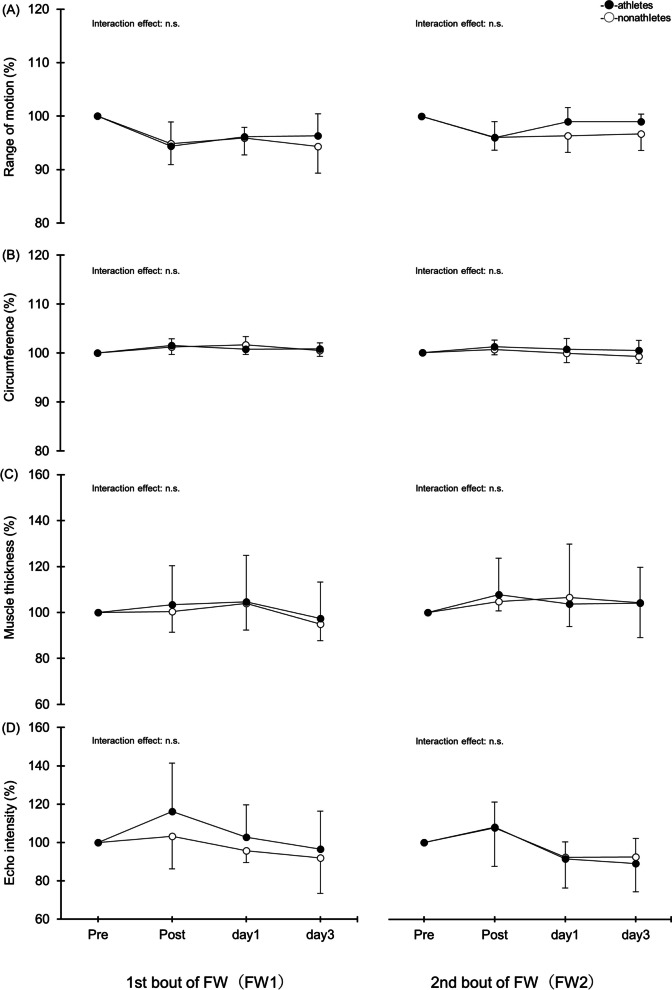
Changes (mean ± SD) in range of motion (**A**), circumference (**B**), muscle thickness (**C**), and echo intensity (**D**) measured before (pre) and immediately after (post) the first (FW1) and second (FW2) flywheel exercises and 1 and 3 days after in the nonathletes and athletes groups

The results for ROM, circumference, muscle thickness, and echo intensity are shown in Fig. 3. There were no significant interactions for these measurements between nonathletes and athletes.

### Comparison between FW1 and FW2

**Fig. 4:**
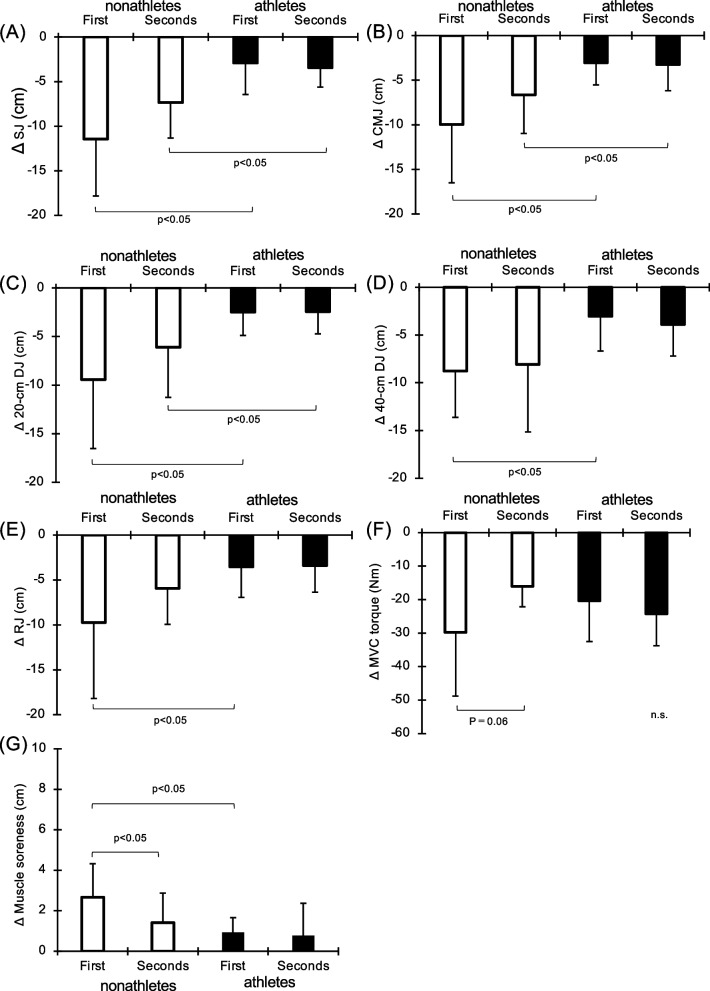
Difference (Δ) from baseline to peak or bottom in squat jump (**A**), countermovement jump (**B**), 20-cm drop jump height (**C**), 40-cm drop jump height (**D**), rebound jump height (**E**), MVC torque (**F**), and muscle soreness (average of vastus lateralis, vastus medialis and rectus femoris muscles) in the first and second flywheel exercise sessions

The differences between the peak values for SJ, CMJ, 20-cm DJ, 40-cm DJ, RJ, MVC torque, and muscle soreness in each group (i.e., the lowest value for SJ, CMJ, 20-cm DJ, 40-cm DJ, RJ, and MVC torque and the highest value for muscle soreness) minus the Pre values are shown in Fig. 4. For SJ, there was no significant difference between FW1 (nonathletes: − 11.4 ± 6.4, athletes: − 2.9 ± 3.6) and FW2 (nonathletes: − 7.3 ± 4.0, athletes: − 3.4 ± 2.2), but there was a significant difference between nonathletes and athletes in FW1 (*p* < 0.05, d = 1.68) and FW2 (*p* < 0.05, d = 1.22). For CMJ, there was no significant difference between FW1 (nonathletes: − 10.0 ± 6.5, athletes: − 3.1 ± 2.5) and FW2 (nonathletes: − 6.7 ± 4.3, athletes: − 3.3 ± 2.9), but there was a significant difference between nonathletes and athletes in FW1 (*p* < 0.05, d = 1.43) and FW2 (*p* < 0.05, d = 0.93). For the 20-cm DJ, there was no significant difference between FW1 (nonathletes: − 9.4 ± 7.1; athletes: − 2.5 ± 2.4) and FW2 (nonathletes: − 6.1 ± 5.1; athletes: − 2.4 ± 2.3), but there was a significant difference between nonathletes and athletes in FW1 (*p* < 0.05, d = 1.34) and FW2 (*p* < 0.05, d = 0.94). For the 40-cm DJ, there was no significant difference between FW1 (nonathletes: − 8.8 ± 4.9; athletes: − 3.0 ± 3.6) and FW2 (nonathletes: − 8.1 ± 7.1; athletes: − 3.9 ± 3.3), but there was a significant difference between nonathletes and athletes in FW1 (*p* < 0.05, d = 1.34). For RJ, there was no significant difference between FW1 (nonathletes: − 9.7 ± 8.4; athletes: − 3.5 ± 3.4) and FW2 (nonathletes: − 5.9 ± 4.0; athletes: − 3.4 ± 3.0), but there was a significant difference between nonathletes and athletes in FW1 (*p* < 0.05, d = 0.98).

In nonathletes, the reduction of MVC torque tended to be smaller (p = 0.06) in FW2 (− 16.0 ± 6.1) than in FW1 (− 29.8 ± 19.0). Meanwhile, there was no significant difference in athletes between FW1 (− 20.3 ± 12.2) and FW2 (− 24.2 ± 9.5). Muscle soreness, which is average value of the vastus lateralis, vastus medialis, and rectus femoris, was significantly smaller in nonathletes in FW2 (1.4 ± 6.1) than in FW1 (2.7 ± 1.7) (*p* < 0.05, d = 0.84). A significant difference was noted between the nonathletes and athletes in FW1 (0.9 ± 0.7) (*p* < 0.05, d = 1.37). No significant difference in athletes was noted between FW1 and FW2 (0.8 ± 1.6).

## Discussion

This study investigated the jump performance and muscle damage after FW exercise and the repeated-bout effect in basketball players and nonathletes. The findings were: (i) the FW exercise resulted in a temporary decrease in jump performance in nonathletes but not in basketball players; (ii) although MVC showed a significant decrease in basketball players, the decrease was less marked than in nonathletes; (iii) the FW exercise resulted in delayed-onset muscle soreness in nonathletes but not in basketball players; and (iv) the repeated-bout effect was confirmed in nonathletes as delayed-onset muscle soreness, while the repeated-bout effect was not found in basketball players. These findings support our hypothesis.

In this study, basketball players showed significantly higher baseline muscle strength, jump performance, and CON peak power due to FW exercise than nonathletes (Table 1). Therefore, the basketball players and nonathletes compared here differed clearly in muscle function and performance. During FW exercise, the CON and ECC peaks and mean power were significantly higher in basketball players than in nonathletes (Table 2). In a previous study, the mean CON peak power before training initiation of university water polo players was 653.2 ± 216.6 W [26]. In the present study, the peak CON and peak ECC power of the basketball players were 886.0 ± 206.7 and 905.6 ± 313.0 W, respectively, so the performance was higher than that in the previous study. Furthermore, although RPE during exercise did not differ between groups, its value was extremely high, showing that the subjects of this study underwent FW exercise with a maximum load (Table 3). However, although ECC power should be greater than CON power, ECC peak and average power were similar to CON peak and average power in athletes. Since the vertical jump of basketball players was higher than that of soccer players [27], we assume that the basketball players are expected to have very high CON power because they perform repetitive jumping movements in their daily basketball practice.

This is the first study to compare changes in jump performance after acute FW exercise among basketball players and nonathletes. Nonathletes showed significant decreases in all tests (SJ, CMJ, DJ, and RJ) after acute FW exercise, whereas basketball players showed no such decreases (Fig. 1). A previous review stated that post-ECC jump performance as a marker of muscle damage decreased significantly at 24‒48 h after exercise [28]. This decrease in jump performance was correlated with an MVC decrease due to knee joint extension (r^2^ = 0.67; *p* < 0.001) [28]. An important point is that the basketball players in the present study showed no decrease in jump performance after FW exercise despite the significant decrease in MVC. As basketball is a sport that involves jump movements approximately every minute [21], the ECCs generated upon landing after a jump routinely exert considerable loads. Therefore, we consider that strong resistance to jump movements is more closely connected to these results than the knee extension model.

MVC decreased significantly in both basketball players and nonathletes after the first FW exercise (Fig. 2). As in a previous study, the FW exercise involving 10 sets of 10 squats performed by young healthy male subjects induced a significant decrease in MVC up to 3 days after the exercise [17]. However, basketball players showed significantly smaller MVC decreases than nonathletes. A previous study comparing muscle damage severity after ECC due to knee joint flexion between resistance-trained and untrained individuals showed that the MVC decrease was significantly smaller in the former [29]. The suggested reason for this difference is that routine sport training results in the repeated-bout effect, while the suggested cause of the smaller decrease in MVC in basketball players in the present study is the repeated-bout effect on the knee extensor muscles.

In the present study, delayed-onset muscle soreness in the vastus lateralis and vastus medialis muscles increased significantly in the nonathletes but did not change significantly in the basketball players (Fig. 2). In previous studies, FW exercises involving squats performed by healthy male subjects resulted in delayed-onset muscle soreness at 1‒3 days after the exercise [16, 17]. However, a study of resistance-trained and untrained subjects found that the decreases in MVC and ROM after ECC by knee joint flexion were significantly lower in resistance-trained subjects, whereas no differences were found in delayed-onset muscle soreness degrees [30]. The suggested reason for this was that delayed-onset muscle soreness is independent of other muscle damage markers. The mechanism for the differences between the results of the present and previous studies is uncertain, but we believe that different types of athletes show different responses to ECCs due to knee joint flexion and delayed-onset muscle soreness due to FW exercise.

In the present study, neither basketball players nor nonathletes showed changes in ROM, circumference, muscle thickness, or echo intensity (Fig. 3). In previous studies, however, ECC due to elbow and knee joint extension and flexion restricted the ROM, while circumference, muscle thickness, and echo intensity increased [24, 31–33]. As far as can be ascertained, no studies to date have evaluated ROM, circumference, muscle thickness, and/or echo intensity after FW exercise. ROM, circumference, muscle thickness, and echo intensity are reportedly dependent upon exercise intensity [34–36]. In the present study, although the maximum exertion achieved in the FW exercise was as high as 10 sets of 10 squats and the RPE was high, the relative load on each muscle may have been lower than that with ECC based on a single-joint exercise. Future studies with multiple load conditions are needed.

Nonathletes displayed an effect of the repeated-bout effect in relation to delayed-onset muscle soreness as well as a tendency in MVC (Fig. 4). In a previous study of nonathlete subjects, the result of FW exercise involving 10 sets of 10 squats performed twice at a 4-week interval showed that, after the second exercise, the MVC decrease, delayed muscle soreness, and serum CK increase were significantly suppressed compared with after the first exercise [17]. The results of the present study support those results, although serum CK was not evaluated. However, in terms of jump performance, for which additional tests were performed in the present study, no repeated-bout effect was noted for any tests (Fig. 4). In a previous study, the result of repeating 50 drop jump exercises after a 2-week interval was that the degree of decrease in SJ and CMJ was significantly lower after the second versus first exercise [37]. In addition, Bridgman et al. [38] found that 50 drop jump exercises induced the repeated-bout effect on jump performance. With respect to the mechanism, multiple factors are complexly involved in the repeated-bout effect, including adaptation of the nerve system and muscle–tendon complexes, remodeling of the extracellular matrix structure, and suppression of the inflammatory response [39]. We speculate that basketball players previously experienced these repeated-bout effects due to routine training. Indeed, in this study, the severity of muscle damage after the first exercise was low in basketball players versus nonathletes, and the repeated-bout effects in basketball players were not seen for any tests (Fig. 4), while a reduction in MVC after the first bout and earlier recovery after the second bout (Fig. 2A). However, the mechanisms connected to the repeated-bout effect on jump performance have not been definitively identified and require clarification in a future study.

## Conclusions

The information obtained in the present study clearly demonstrates that intense and acute FW exercises involving squat movements did not reduce the jump performance of basketball players. Additionally, although basketball players showed decreased muscle strength, they did not develop delayed-onset muscle soreness. Although the FW exercise reduced jump performance and caused muscle damage in nonathletes, our findings suggest the presence of repeated-bout effects on delayed-onset muscle soreness and muscle strength. These findings indicate that basketball players could add FW exercises to their training routine if they consider the reduction in muscular function that will occur after the first FW session, while nonathletes require recovery periods. Earlier recovery was seen after the second than first session, especially in nonathletes. We believe that this information may be useful for preparing FW training programs for basketball players.

## Data Availability

The datasets created during the current study are available from the corresponding author upon reasonable request.
